# Microwave-Assisted Fabrication of Mesoporous Silica-Calcium Phosphate Composites for Dental Application

**DOI:** 10.3390/polym13010053

**Published:** 2020-12-25

**Authors:** Adrian Szewczyk, Adrianna Skwira, Marta Ginter, Donata Tajer, Magdalena Prokopowicz

**Affiliations:** 1 Department of Physical Chemistry, Faculty of Pharmacy, Medical University of Gdańsk, Hallera 107, 80-416 Gdańsk, Poland; adrian.szewczyk@gumed.edu.pl (A.S.); adrianna.skwira@gumed.edu.pl (A.S.); martaginter@gumed.edu.pl (M.G.); donatasmentoch@gmail.com (D.T.); 2 Scientific Circle of Students, Department of Physical Chemistry, Faculty of Pharmacy, Medical University of Gdańsk, Hallera 107, 80-416 Gdańsk, Polland

**Keywords:** mesoporous silica, calcium orthophosphates, hydroxyapatite, composites, rapid coating, simulated body fluid, mineralization

## Abstract

Herein, the microwave-assisted wet precipitation method was used to obtain materials consisting of mesoporous silica (SBA-15) and calcium orthophosphates (CaP). Composites were prepared through immersion of mesoporous silica in different calcification coating solutions and then exposed to microwave radiation. The composites were characterized in terms of molecular structure, crystallinity, morphology, chemical composition, and mineralization potential by Fourier-transform infrared spectroscopy (FTIR), powder X-ray diffraction (XRD), and scanning electron microscopy equipped with energy-dispersive X-ray spectroscopy (SEM-EDX). The application of microwave irradiation resulted in the formation of different types of calcium orthophosphates such as calcium deficient hydroxyapatite (CDHA), octacalcium phosphate (OCP), and amorphous calcium phosphate (ACP) on the SBA-15 surface, depending on the type of coating solution. The composites for which the progressive formation of hydroxyapatite during incubation in simulated body fluid was observed were further used in the production of final pharmaceutical forms: membranes, granules, and pellets. All of the obtained pharmaceutical forms preserved mineralization properties.

## 1. Introduction

Presently, synthetic and natural polymers make a significant contribution to the fabrication of restorative biomaterials and drug carriers in the field of dentistry [1]. Among them, organic polymers such as polymethyl methacrylate (PMMA), polyacrylamides, polyacrylates, polyethylene glycol (PEG), polyurethane (PUR), polylactic acid (PLLA), collagen, chitosan, and celluloses have been widely used as cements [2,3], resins [4,5], scaffolds [6,7], membranes [8,9], gels [10,11], sponges [12,13], and fibers [14,15].

However, not only organic but also inorganic polymers such as mesoporous silica (MS) have been investigated in terms of dental application. More and more studies have emphasized the potential application of MS in dentistry, due to its unique properties such as ordered pore arrangement and narrow pore size, high specific surface area, modifiable shape, thermal resistance, simple surface functionalization, and drug loading capacity. In studies concerning mechanical properties of dental biomaterials, MS has been investigated as a resin-reinforcing agent [16,17,18,19]. Samuel et al. [18] have claimed that a combination of MS with nonporous silica resulted in both hydrolysis- and wear-resistant dental composites, and Martim et al. [19] proved that MS-containing resins were characterized by greater resistance to leaching unreacted monomers after being immersed in an organic solvent. Considering the studies on restorative dental biomaterials, MS-containing biomaterials are examined in terms of so-called biomimetic mineralization properties defined as a self-formation of surface hydroxyapatite with morphology and composition similar to natural apatite [20]. Such self-formed surface hydroxyapatite is widely known as a fully biocompatible and osteoconductive interface between the implanted biomaterial and surrounding tissue [21]. Hence, the addition of MS seems to accelerate the mineralization process due to the presence of silanol groups, which promote the hydroxyapatite nucleation [22]. In both in vitro and in vivo studies conducted by Chiang et al. [23,24], calcium carbonate-loaded MS mixed with phosphoric acid led to mineralization of microporous dentin structures with dentinal tubules occluded by biomimetic hydroxyapatite precipitates. Additionally, Canto et al. [25,26] have claimed that calcium-loaded MS can prevent the progression of enamel erosion, likely due to the capacity of the silica to increase the bioavailability of incorporated molecules, thus supporting the mineralization process. 

The full covering of the MS surface by self-formed hydroxyapatite in vitro typically ranges from 30 to 60 days [27,28]. Thus, to enhance the mineralization properties of MS-containing polymeric biomaterials, the addition of calcium phosphates has been widely explored [29,30], with particular attention paid to synthetic and natural hydroxyapatites [31,32,33]. In vivo investigations confirmed that hydroxyapatite-containing biomaterials exhibit both osteoinductive and osteoconductive properties and provide the connection between the implant and alveolar bone [34] or tooth [35]. In addition, the use of biomimetic hydroxyapatite provides significant enamel remineralization after its application [36]. Various methods of hydroxyapatite synthesis, providing similar composites of mesoporous silica and calcium phosphate, have been reported (cementitious conversion of α-tricalcium phosphate to hydroxyapatite in the presence of SBA-15 [37]; in situ precipitation of hydroxyapatite in the TiO_2_/SBA-15 via facile inner-pore sol-gel method [38]; hydroxyapatite crystals growth inside the pores of Ca-doped SBA-15 matrix after its immersion in (NH_4_)_2_HPO_4_ solution [39]); however, microwave-assisted wet precipitation was found to be more economic, efficient, and faster than conventional routes of synthesis [40,41,42]. Moreover, the usage of microwave radiation results in hydroxyapatite particles characterized by structure and composition similar to the biological one [43].

In this work, we obtained mesoporous silica-calcium phosphate composites using a simple microwave-assisted wet precipitation method. For this purpose, mesoporous silica SBA-15 was chosen as an initial material due to its confirmed application potential in dentistry (resins-reinforcing agent [18] and cytocompatible filler [44], tooth remineralization enhancing agent [45], drug carrier [46]). The obtained SBA-15 material was next suspended in rapid coating solutions dedicated to hydroxyapatite precipitation that were selected based on a literature review. Suspensions were exposed to microwave radiation treatment in order to obtain SBA-15-calcium phosphate (SBA-CaP) composites. In the next step, obtained composites were examined in terms of mineralization properties in simulated body fluid. 

Finally, the SBA-CaP composite characterized by the highest mineralization properties was used in the manufacturing process of membranes, granules, and pellets (spherical granules with increased mechanical resistance). During the manufacturing process of the above-mentioned pharmaceutical forms, polymers such as microcrystalline cellulose (MCC), ethyl cellulose (EC), and polydimethylsiloxane (PDMS) were used as excipients. The excipients were chosen based on their confirmed safety in dentistry [47,48,49]. Each pharmaceutical form was next immersed in simulated body fluid to verify whether the SBA-CaP composite preserved mineralization properties after the manufacturing process. 

## 2. Materials and Methods 

### 2.1. Synthesis of Mesoporous Silica SBA-15

The mesoporous silica SBA-15 was synthesized via sol-gel method [50] using tetraethyl orthosilicate (TEOS, Sigma-Aldrich, Poznan, Poland) as a silica precursor, triblock copolymer poly(ethylene oxide)-poly(propylene oxide)-poly(ethylene oxide) (Pluronic P123, Mn = 5800, Sigma-Aldrich) as a template, and 2 M HCl as a catalyst. Pluronic P123 (4.0 g) was dissolved in the mixture containing 120 g of 2 M HCl and 30 g of purified water using a magnetic stirrer (300 rpm, 35 °C). Next, 8.5 g of TEOS was added, and the mixture was stirred for 20 h under the same conditions. The obtained suspension was aged (80 °C, 24 h), filtered, and calcined at 500 °C using a muffle furnace (FCF 7SM, Czylok, Jastrzebie-Zdroj, Poland, 1 °C/min heating rate). The obtained powder was homogenized in a mortar, sieved, and stored in a desiccator. For further studies, silica powder with a particle size of 200–500 µm was used to provide homogenous dispersion of SBA-15 material after immersion in rapid-coating solutions.

### 2.2. Preparation of Coating Solutions

Different supersaturated calcification coating solutions with high calcium and phosphate ion concentrations were prepared, based on the literature review [51,52,53,54]. For comparative purposes, conventional simulated body fluid (SBF) with an ion concentration close to that of human blood plasma was prepared [55]. Each solution was prepared according to the reference (Table 1). Briefly, chemicals required for each solution were added one by one and completely dissolved in 800 mL of purified water at room temperature. Next, the volume of each solution was adjusted to 1 L using a volumetric flask. All solutions were prepared shortly before studies. The chemicals sodium chloride (NaCl), potassium chloride (KCl), sodium hydrogen carbonate (NaHCO_3_), calcium chloride dihydrate (CaCl_2_·2H_2_O), magnesium chloride hexahydrate (MgCl_2_·6H_2_O), sodium sulfate (Na_2_SO_4_), sodium dihydrogen phosphate monohydrate (NaH_2_PO_4_·H_2_O), and potassium hydrogen phosphate trihydrate (K_2_HPO_4_·3H_2_O) were used as received (>99.0% purity, analytical grade, Sigma-Aldrich). The ion concentrations of each solution are presented in Table 1.

### 2.3. Preparation of SBA-15-CaP Composites

To obtain SBA-15-calcium phosphate (SBA-CaP) composites, 100 mg of SBA-15 powder (200–500 µm) was suspended in 100 mL of each prepared solution and exposed to microwave irradiation using 9 cycles of 30s microwave heating at 600 W for each 100 mL of solution (microwave oven, Series 2 HMT84, BOSCH, Gdansk, Poland) with 30s break between cycles. The suspensions were left for 24 h in a water bath (Witeg WSB-30, 75 rpm, 37 °C). For comparative purposes, the SBA-CaP composites were also obtained by standard coating procedure, in which SBA-15 was suspended in prepared solutions without exposure to microwave irradiation. Next, all suspensions were filtered, and obtained composites in the form of powders were dried at 90 °C overnight and homogenized using sieving to obtain a 200–500 µm particle size. The 200–500 μm particle size provided a homogenous dispersion of composites in SBF during mineralization properties assay (Section 2.4) and repeatable distribution of composites in the formulation of pharmaceutical forms (Section 2.5). The obtained SBA-CaP composites were named as SBA-CaP(MW)_Sx and SBA-CaP(S)_Sx for microwave-assisted and standard coating procedures, respectively, where x corresponds to the number of used coating solution (S1–S5). 

Then, composites were analyzed in terms of Fourier-transform infrared spectroscopy (FTIR), powder X-ray diffraction (XRD), and scanning electron microscopy equipped with energy-dispersive X-ray spectroscopy (SEM-EDX). The FTIR, XRD, and EDX analyses were carried out to determine the type of calcium phosphate formed in the obtained composites. The SEM analysis was applied to find out if the SBA-15 material was coated with formed calcium phosphate. Only the samples for which the calcium phosphate coating on SBA-15 surface was observed (SBA-CaP(MW)_S1-S4) were further investigated in terms of mineralization properties assay. The flowchart of the study design was presented in Supplementary Material (Figure S1). 

### 2.4. Mineralization Properties Assay of SBA-CaP Composites

The SBA-CaP(MW)_S1-S4 composites were studied using one of the most popular simulated body fluids proposed by Kokubo and Takadama [55]. Each composite was immersed for 7 days in SBF using a 1:1 (m/*v*) ratio between composite and SBF. Briefly, 100 mg of composites was immersed in 100 mL of SBF in a polypropylene flask. The experiment was performed in a water bath (Witeg WSB-30) at 37.0 °C under stirring conditions (70 rpm). The SBF was exchanged every 24 h by simple decantation method. After 7 days of mineralization study, composites were filtered, rinsed with 50 mL of purified water, dried at 40 °C, and analyzed using FTIR, XRD, and SEM-EDX methods. Then, composites that showed mineralization properties (SBA-CaP(MW)_S1, S2, S4), manifested by observed calcium phosphate clusters on SBA-15 materials (SEM), changes in Ca/P molar ratio (EDX), increased intensity of P-O bands in FTIR, and new peaks of hydroxyapatite phase in XRD, were immersed in SBF for the next 7 and 14 days to observe the progressive surface hydroxyapatite formation using the above-mentioned analytical methods.

### 2.5. Formulation of Pharmaceutical Forms

The SBA-CaP composite that showed the highest mineralization properties was used to obtain pharmaceutical forms of membranes, granules, and pellets. The membranes were obtained using the solvent-evaporation molding method proposed in our previous studies [56,57]. Briefly, membranes were prepared by mixing 5% (*w*/*w*) ethyl cellulose (EC, Aqualon N22 Pharm, 20 cP, Ashland, Warsaw, Poland) in 95% (*v*/*v*) ethanol with 2% (*v*/*v*) addition of hydroxyl-terminated polydimethylsiloxane (PDMS, 65 cSt, Sigma-Aldrich) and 6 mg addition of SBA-CaP composite per 250 µL of a blend. The prepared coating blends were homogenized by using sonication in a cooling bath for 20 min. Equal 250 µL volumes of the blend were poured into polypropylene molds and incubated till complete ethanol evaporation (30 °C, 60% relative humidity, 24 h). The end of the solidification of the polymer membranes was regarded as the moment when no changes in weight were detected (within the limits of instrumental error (±0.01 g)).

Granules were obtained using sieving as the traditional wet granulation method. The SBA-CaP composite was wetted in a mortar using a binder composed of 5% (*w*/*w*) EC in 95% (*v*/*v*) ethanol with 2% (*v*/*v*) addition of PDMS providing a 1:2.5 m/m ratio between powder and binder. Next, a wet mass was forced through a 500 µm sieve, and obtained granules were dried at 40 °C for 24 h.

The pellets were prepared using the wet granulation, extrusion, and spheronization process in the Caleva Multi Lab apparatus (5 g batch size). The operating conditions were optimized in preliminary studies. First, 2.5 g of SBA-CaP composite, together with 2.25 g of microcrystalline cellulose (MCC, Avicel PH 101, Sigma-Aldrich), and 0.25 g of EC were mixed in a granulator attachment (100 rpm, 2 min). Next, 11.0 g of binder (the same as used in the preparation of granules) was added, and powders were mixed for 5 min. Obtained wetted mass was extruded in an extruder attachment (80 rpm, 1 mm holes). The entire batch of the extrudate was spheronized in a spheronizer attachment (2000 rpm, 5 min). The final pellets were left to dry at 40 °C for 24 h.

The obtained pharmaceutical forms were examined in terms of the mineralization properties in SBF. The studies were carried out following a similar procedure as described for SBA-CaP composites, except that a 2:1 (m/v) ratio between pharmaceutical form and SBF was provided. Each form was soaked in SBF for 21 days with SBF exchange every 24 h. Then, forms were rinsed with 50 mL of purified water, dried, and examined using the SEM-EDX and FTIR method. Due to the relatively low mineralization potential of obtained membranes (Appendix A), they were additionally subjected to the microwave-assisted rapid coating procedure using S1 coating solution, rinsed with 50 mL of purified water, dried, and once again investigated in SBF for 21 days. This procedure was not necessary for granules and pellets, which exhibited great mineralization properties right after the manufacturing process.

### 2.6. Characterization Methods

#### 2.6.1. FTIR

Samples were characterized using a Fourier-transform infrared spectroscopy in the range of 4000 cm^−1^–400 cm^−1^ (FTIR, Jasco model 410, Pfungstadt, Germany, 4 cm^−1^ resolution) using the potassium bromide (KBr) disk technique. Each 1 mg sample was mixed with 100 mg of KBr, compressed into the disk, and analyzed. For comparison purposes, the FTIR spectra were normalized to maximum absorption of the dominant peak at ∼1080 cm^−1^.

#### 2.6.2. XRD

Wide-angle X-ray powder diffraction (XRD) data were recorded on the Empyrean PANalytical diffractometer using CuKα radiation (40 kV and 25 mA) at a scanning rate of 1 deg/min with a step width of 0.02 in the 2θ range of 5–60. The 5 mg samples were used in each analysis. 

#### 2.6.3. SEM-EDX

The morphology and the surfaces of the samples were examined using scanning electron microscopy combined with X-ray energy dispersive spectrometry (SEM-EDX; Hitachi SU-70, Japan and Quanta 3D FEG, Poland). Three samples of obtained composites and pharmaceutical forms were independently investigated, choosing five random sites of interest each time at × 350, 1000, or 5000 magnification. For powders and granules, each sample mass was approx. 2 mg. For pellets and membranes, each sample included one individual pharmaceutical form. All prepared samples were coated with a 10 nm gold layer and analyzed using 3.0–20.0 kV operating voltage. The Ca/P molar ratio was expressed as a mean value ±SD calculated from five independent EDX measurements. The size of observed CaP specimens was expressed as a mean value from ten measurements.

#### 2.6.4. Stereoscopic Microscopy

The overall shape of obtained pharmaceutical forms was investigated using microscope Opta-TECH X 2000 (Warsaw, Poland, plan-achromatic optic, parallel optical path).

## 3. Results and Discussion

**Figure 1:**
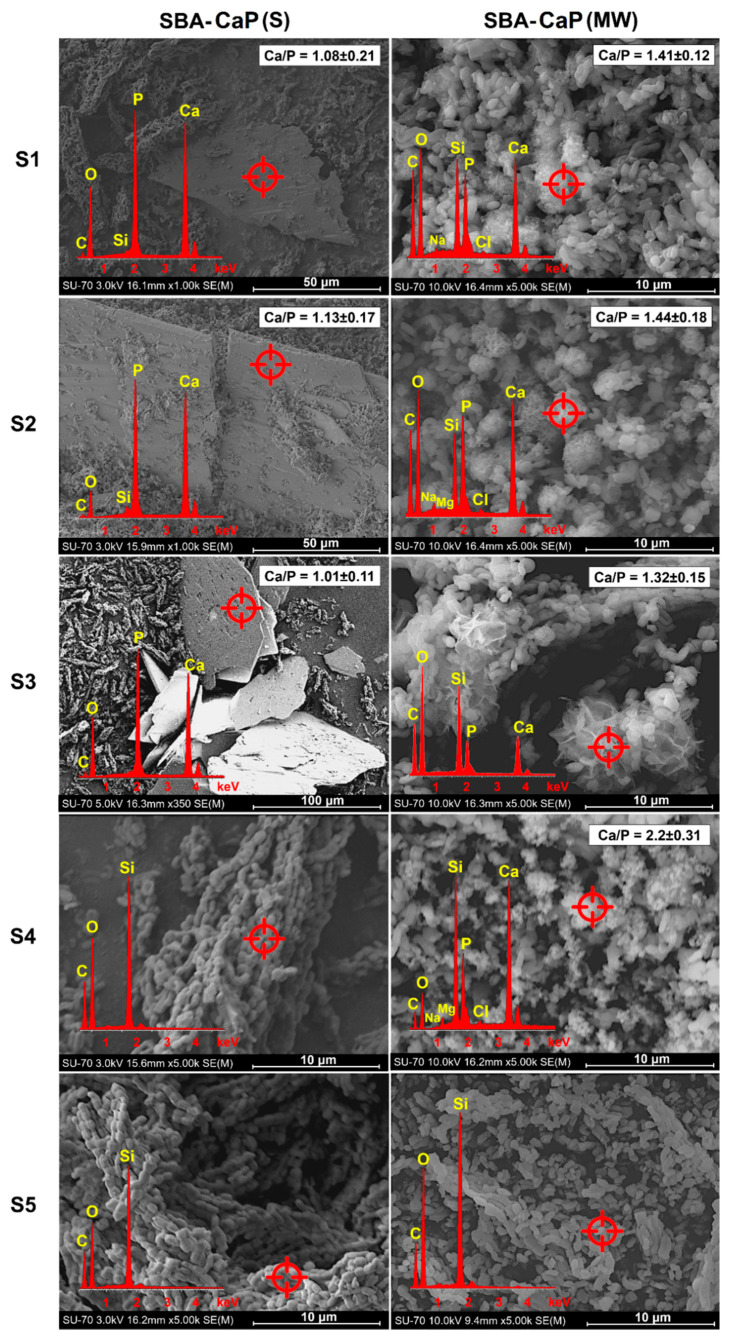
SEM-EDX micrographs of silica-calcium phosphate composites obtained using standard (SBA-CaP(S)) and microwave-assisted (SBA-CaP(MW)) rapid coating procedures with (**S1**–**S5**) coating solutions.

The SEM-EDX micrographs of both SBA-CaP(S) and SBA-CaP(MW) samples are presented in Figure 1. Each sample revealed the characteristic fiber-like morphology of synthesized SBA-15 silica rods (as reported in our previous studies [27,58], such obtained SBA-15 silica rods were characterized by well-ordered hexagonal mesopores (~7 nm diameter) and specific surface area above 600 m^2^/g). The use of conventional rapid coating procedure without microwave irradiation resulted in either precipitation of calcium phosphate crystals (50 μm–100 μm size) with platelet morphology (SBA-CaP(S)_S1-S3) or lack of precipitation (SBA-CaP(S)_S4-S5). Basing on the observed morphology and Ca/P molar ratio ~1.0, the presented type of precipitated calcium phosphate was characterized as dicalcium phosphate dihydrate (DCPD) [59,60]. Additionally, pH of S1-S3 solutions varied within a range from 6.3 to 6.5, which also confirmed the acceleration of DCPD precipitation [61,62]. Moreover, Lu et Leng [63] have concluded that DCPD is the most kinetically favorable phase that precipitates in SBF when the concentrations of both calcium and phosphate exceed the normal level of conventional SBF. The FTIR and XRD analyses of SBA-CaP(S)_S1-S3 samples also confirmed the presence of DCPD in the composites (Supplementary Material Figure S2). Both the sharp peaks in XRD patterns and the well-split phosphate bands of HPO_4_^2−^ groups [64] were connected with the crystalline DCPD phase. As well as the SEM-EDX results, the screening FTIR and XRD analyses did not confirm the presence of CaP in SBA-CaP(S)_S4 and S5 samples. Due to the fact that SBA-15 surface was not coated with calcium phosphate during the standard rapid coating procedure using S1–S5 solution (only a separate phase precipitated DCPD crystals in SBA-CaP(S)_S1–S3 composites was observed), the obtained composites were not further investigated according to the flowchart of the study design (Figure S1).

Contrary to the standard rapid coating procedure, the use of microwave-assisted procedure resulted in three different types of calcium phosphate precipitates heterogeneously formed on the SBA-15 surface (Figure 1). The heterogeneous nature of calcium phosphate precipitation on biomaterial surface is a well-known phenomenon that promotes further biomineralization [52,65,66,67]. For SBA-CaP(MW)_S1-S2 samples, a calcium-deficient hydroxyapatite (CDHA) was detected with so-called cauliflower morphology [68] and approx. 1.42 Ca/P molar ratio, whereas for SBA-CaP(MW)_S3 sample, the plate-like particles of octacalcium phosphate (OCP) [60,69] with adequate Ca/P ~1.32 were observed. Furthermore, the amorphous calcium phosphate (ACP) [70] was formed as the spherical particles (Ca/P ~2.2) randomly packed in the clusters around SBA-15 (SBA-CaP(MW)_S4 sample). No precipitates of calcium phosphate were observed on the SBA-15 surface after 24h immersion in conventional SBF solution after microwave irradiation (SBA-CaP(MW)_S5). The FTIR and XRD screening analyses (Figure S2) were consistent with presented SEM-EDX results and confirmed the presence of calcium phosphates in composite (characteristic vibrational modes of PO_4_^3−^ groups at 560 cm^−1^–600 cm^−1^ and XRD patterns in the ~30° 2θ range). Moreover, no presence of DCPD was noticed in all SBA-CaP(MW) composites. Thus, SBA-CaP(MW)_S1-S4 composites were further investigated in terms of mineralization properties assay.

The influence of microwave irradiation on calcium phosphate formation in rapid coating solutions seemed to be crucial and might be explained as follows. The temperature of all investigated solutions increased from 25 °C to 90 °C during irradiation and thus reduced the kinetic barriers of ACP, OCP, and CDHA synthesis [40]. It should be noted that the microwave-assisted synthesis of hydroxyapatites was found to be faster and more efficient compared to conventional heating, for which the obtaining of stoichiometric hydroxyapatite was only possible at a temperature >100 °C [40]. Moreover, the microwave irradiation can reduce the hydration spheres around calcium ions through the absorption of microwave energy by water molecules. Locally bared calcium ions accelerate the ionic interaction with phosphates and hydroxyl groups [40,43], which leads to the formation of more complex structures than DCPD crystals. Additionally, the presence of SBA-15 silica in rapid coating solutions should be also considered. The residual silanol groups (Si–OH) present on the SBA-15 surface act as the nucleation centers for hydroxyapatite formation [28,58]. Thus, the SBA-15 surface might act as a preferable site for in situ formation of calcium phosphates during the microwave-assisted rapid coating procedure. Furthermore, the influence of coating solution composition on the type of calcium phosphate formed on SBA-15 surface during microwave irradiation cannot be neglected and will be explained in detail after additional studies. 

**Figure 2:**
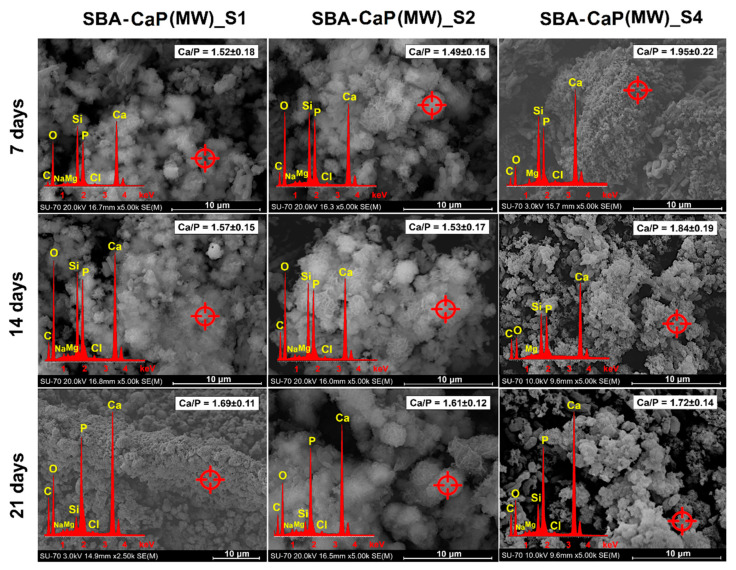
SEM-EDX micrographs of selected SBA-CaP(MW) composites after 7 days, 14 days, and 21 days of immersion in simulated body fluid.

The SEM-EDX results of the progressive formation of hydroxyapatite on the surfaces of SBA-CaP(MW)_S1, S2, and S4 composites in SBF are presented in Figure 2. Due to the poor mineralization properties of SBA-CaP(MW)_S3 composite after 7 days of incubation in SBF, the SEM-EDX data together with XRD and FTIR for this composite are presented in Supplementary Material (Figure S3). 

The formation of the apatite layer on the composite’s surface goes through a sequence of chemical reactions such as precipitation, nucleation, and growth of calcium phosphate [71]. For both SBA-CaP(MW)_S1 and S2 composites, CDHA particles created larger clusters that progressively spread all over the silica. After 21 days of incubation in SBF, SBA-15 silica rods in both composites were completely covered by the continuous layer of hydroxyapatite. This observation was further confirmed by the EDX results. As a function of immersion time in SBF, the Ca/P molar ratio increased from ~1.50 (characteristic for CDHA) to ~1.65 (characteristic for HA). A similar observation was reported by Xiong et al. [72], who detected apatite layer (Ca/P = 1.63) on CDHA-poly(amino acid) composite after 16 weeks of incubation in SBF. This phenomenon might be related to the partial dissolution of CDHA in SBF with its simultaneous re-deposition in the form of HA as a consequence of released calcium and phosphate ions. In fact, Li et al. [73] claimed that the presence of alginate-stabilized CDHA in the biphasic calcium phosphate-based ceramic provided greater nucleation points for the deposition of bone-like apatite after immersion in SBF than conventional ceramic. For SBA-CaP(MW)_S4 sample (Figure 2), the gradual transformation of ACP from spherical particles into the hydroxyapatite with plate-like morphology was observed. 

Similar findings were reported by Fu et al. [74] and Zhou et al. [75], who obtained ACP-contained polylactic acid nanofibers or modified tantalum scaffolds for bone regeneration, respectively. In both cases, the plate-like particles of hydroxyapatite after immersion in SBF were formed on investigated composites, providing osteoconductive properties in vitro and in vivo. Therefore, two different mechanisms of hydroxyapatite formation in SBF might be observed for obtained composites: first, based on progressive dissolution/re-deposition of DCHA (SBA-CaP(MW)_S1-S2 sample) and second, related to the transformation of ACP into the apatite (SBA-CaP(MW)_S4 sample). Nonetheless, in both cases, the presence of silanol groups on the SBA-15 surface supported the apatite formation, which was proven in previous studies [27,28,58].

**Figure 3:**
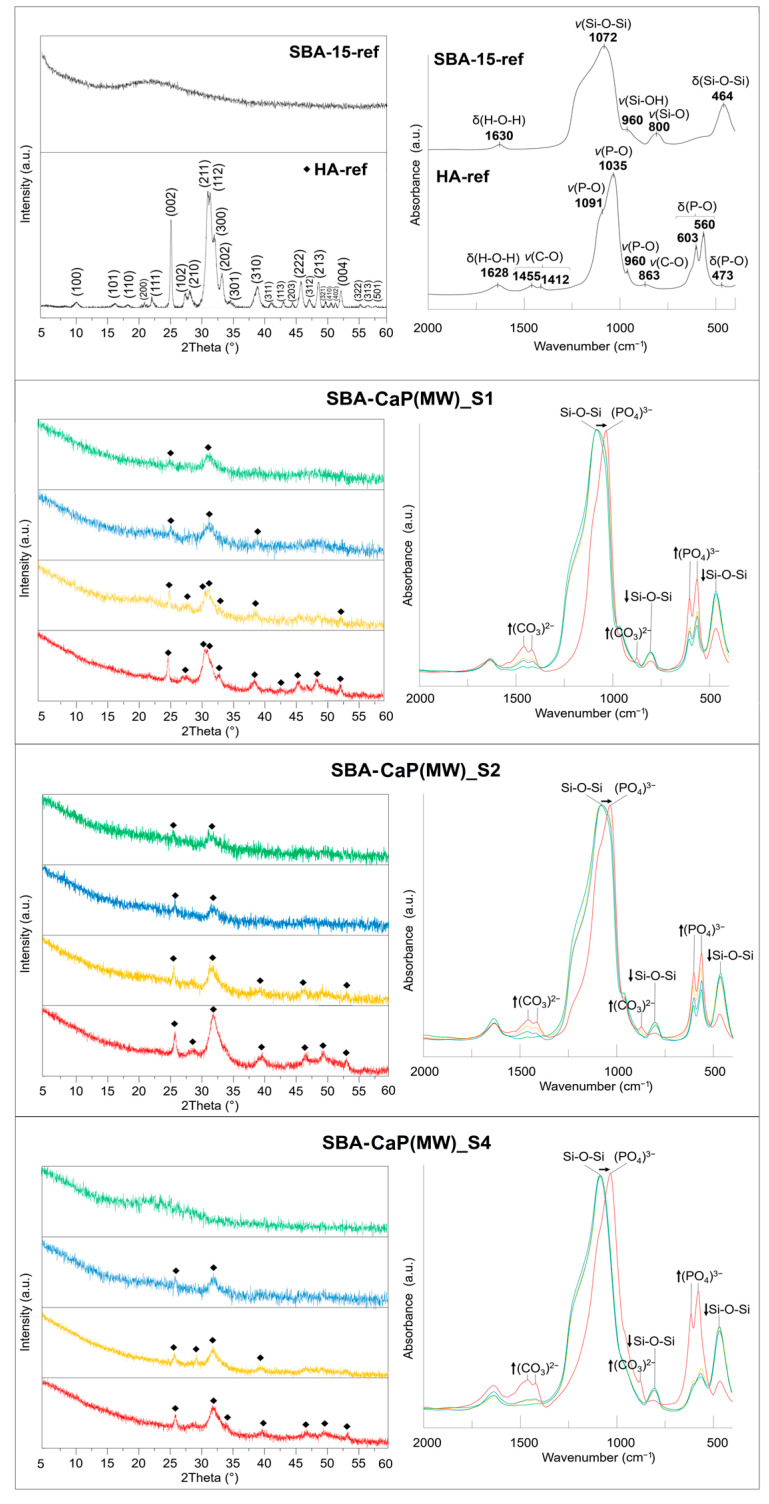
The XRD (**left**) and FTIR (**right**) results of selected SBA-CaP(MW) composites before (**green**) and after 7 (**blue**), 14 (**yellow**), and 21 (**red**) days of incubation in simulated body fluid together with SBA-15 and HA reference samples (types of vibration: ν—stretching, δ—bending).

The progressive formation of hydroxyapatite on the composites’ surface was also confirmed using XRD and FTIR methods (Figure 3). Before immersion in simulated body fluid, the XRD diffractograms (green line) of SBA-CaP(MW)_S1 and S2 composites revealed a poorly crystalline hydroxyapatite phase characterized by (002) and (211) peaks, whereas for the SBA-CaP(MW)_S4 sample, the amorphous nature of the calcium phosphate phase was proved in agreement with SEM-EDX results (Figure 1 and Figure 2). However, on the surface of all investigated composites, the semi-crystalline hydroxyapatite phase was gradually formed as a function of incubation time in SBF, which was confirmed by the appearance of additional peaks characteristic for hydroxyapatite ((210), (310), (222), (213), (004)). Less evident peaks (112) and (113) could be only seen for the SBA-CaP(MW)_S1 sample. When the soaking time of composites in SBF increased, the diffractograms of the formed apatite phase became sharper, proving the progressive transformation and crystallization of obtained calcium phosphates into the hydroxyapatite. The FTIR spectra of obtained composites (green line) revealed bands characteristic of both SBA-15 silica and calcium phosphate. The bands at ~1070 cm^−1^, 800 cm^−1^, and 460 cm^−1^ were attributed to asymmetric, symmetric stretching, and deformative vibrations of Si-O, respectively [76]. Interestingly, the band characteristic for silanols at 960 cm^−1^ was either not present or characterized by the low intensity in all investigated samples, which suggested the interaction between free silanols and calcium phosphate during the microwave-assisted rapid coating procedure. The bands at ~600 cm^−1^ and 560 cm^−1^ observed in both SBA-CaP(MW)_S1 and S2 spectra were connected with ν4 vibrational mode of P-O bond in CDHA [77]. The broad band at 560 cm^−1^ present in SBA-CaP(MW)_S4 was characteristic for ACP [70]. During the composites; incubation in SBF, the reduction of bands intensity characteristic for silica with a simultaneous increase in bands intensity of PO_4_^3−^ groups showed the progressive formation of hydroxyapatite layer on composites surface. Moreover, the shift of bands at maximum absorbance from ~1070 cm^−1^ to ~1040 cm^−1^ (after 21 days of incubation in SBF) proved the dominant contribution of hydroxyapatite bonds in the investigated composites. The additional increase in bands’ intensity characteristic for CO_3_^2−^ groups at ~1430 cm^−1^ and ~860 cm^−1^ was noted; thus, incorporation of carbonates into the hydroxyapatite structure occurred, which is a well-known phenomenon in SBF [58,77,78]. The above-mentioned observations of both XRD and FTIR results confirmed the mineralization potential of the obtained composites as well as the SEM-EDX micrographs (Figure 2). 

**Figure 4:**
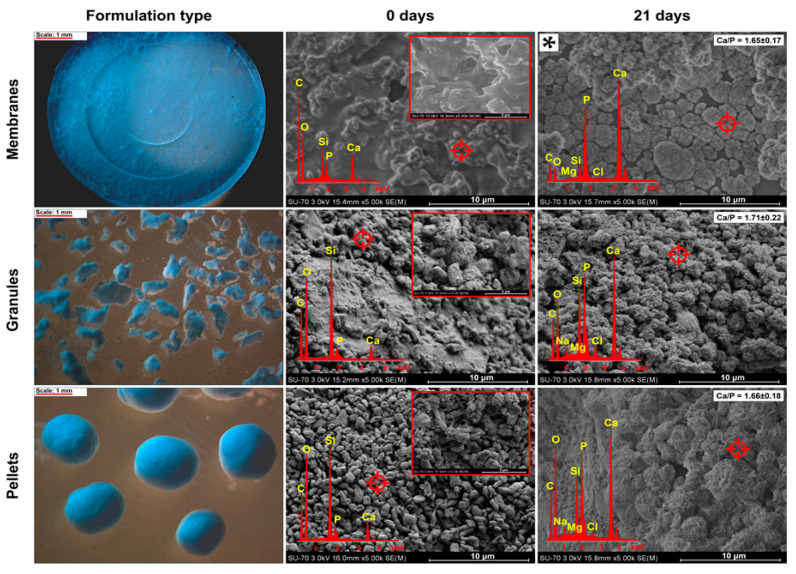
Stereoscopic and SEM-EDX micrographs of obtained pharmaceutical forms before and after 21 days of incubation in simulated body fluid. * The micrograph of sample subjected to additional rapid coating procedure before 21-day incubation in simulated body fluid.

Based on the obtained results, the SBA-CaP(MW)_S1 composite was used in the formulation of membranes, granules, and pellets due to its relatively high mineralization properties. The micrographs of obtained pharmaceutical forms are presented in Figure 4. 

All investigated pharmaceutical forms were characterized by satisfactory mechanical properties—no disintegration during mineralization properties assay was observed. The obtained solid membranes were characterized by a compact and heterogeneous surface: the particles of SBA-CaP(MW) composite were suspended in a continuous layer of ethyl cellulose. After 21 days of membranes incubation in SBF, a thin layer of CDHA clusters was formed with approx. 1.51 Ca/P molar ratio (Appendix A). This observation might be explained as a consequence of ethyl cellule presence, which acted as a hydrophobic barrier that impeded the ion exchange between SBA-CaP composite and SBF solution. A similar finding was reported by Hokmabad et al. [79], who observed that after 14 days of incubation in SBF, the mineralization potential of ethyl cellulose-poly (ε-caprolactone)-alginate scaffolds without at least 10% addition of HA was negligible. Therefore, to increase the mineralization potential, the obtained parent membranes were additionally subjected to microwave-assisted rapid coating procedure and immersed in SBF for 21 days. After such treatment, a continuous layer of biomimetic apatite was formed on the membranes’ surface (Ca/P~1.70), providing excellent mineralization properties (Figure 4). 

The microscopic image of obtained granules (Figure 4) showed fine, 200–500 μm, agglomerates of SBA-CaP(MW) composite bounded with ethyl cellulose-PDMS blend, whereas the manufactured pellets were characterized by spherical shape and particle size in the range of 0.8–1.0 mm. SEM micrographs of both granules and pellets revealed their main components: SBA-15 particles together with CDHA (Figure 4, inset) and ethyl or microcrystalline cellulose domains. After 21 days of incubation in SBF, the granule’s surface was covered by a continuous layer of HA; however, some of the ethyl cellulose-rich areas were less coated by HA due to the low mineralization properties of bare celluloses. Similar observations were reported for pellets: favorable formation of HA was noted in SBA-CaP(MW)-rich regions. The progressive formation of HA on the surfaces of each obtained pharmaceutical form has been also confirmed using FTIR analysis (Appendix B). Therefore, the proposed pharmaceutical forms exhibited the mineralization properties in SBF characterized by SEM-EDX and FTIR results (Figure 4, Appendix B), in agreement with corresponding analyses for parent SBA-CaP(MW)_S1 composite (Figure 2 and Figure 3).

The presented in vitro results of mineralization properties assay provide a solid basis for further investigations, including the in vivo studies. For example, Chiang et al. [24] proposed a calcium carbonate-mesoporous silica composite that rapidly formed CaHPO_4_ after its mixing with phosphoric acid. The obtained paste was next implanted inside the tooth of mongrel dogs and exhibited excellent mineralization properties providing the formation of DCPD, OCP, α-TCP, and HA, which acted as promoters for the pulp-dentin complex mineralization. Other researchers have also emphasized the clinical potential of bioactive mesoporous silica-calcium phosphate composites [37,39]. Moreover, the materials intended for dental application should not only promote the remineralization of tissue but also suppress the bacterial biofilm formation [4]. According to the confirmed in vitro mineralization properties of presented SBA-CaP composites and previously reported high drug loading capacity of mesoporous silica-CaP materials together with prolonged release profiles [78], the composites may be considered as a promising dual-function additive to dental resins. Zhang et al. [80] proposed a dental resin enriched with calcium-doped and ciprofloxacin-adsorbed mesoporous silica as a restorative material. In another study [81], collagen fibrils with ACP-loaded mesoporous silica were proposed as bone/teeth mineralizing scaffold with the mentioned drug-loading capacity. In both cases, the fabrication of final composites was a multistage process that seems to make their production difficult on a larger scale. Due to the fact that proposed SBA-CaP composites exist in the form of powder, they can be easily transformed in pharmaceutical process into the final form with shape dedicated for specific use. Thus, various dental drug delivery systems might be produced using the same mineralization agent. However, additional studies, including drug loading capacity, pharmaceutical availability, and biocompatibility tests must be carried out to better examine the application potential of proposed composites.

## 4. Conclusions

In this study, mesoporous silica-calcium phosphate composites characterized by high mineralization potential were successfully obtained via microwave-assisted rapid coating method. The type of calcium phosphate formed on the SBA-15 surface is determined by the formulation of the coating solution used. Therefore, it suggests that the proposed microwave-assisted rapid coating method is not only fast and efficient but also highly manageable. The conducted experiments of mineralization properties showed that the mechanism of hydroxyapatite formation during the soaking of final composites in SBF for 21 days, its morphology, and its crystallinity depend on the type of calcium phosphate initially presented on the SBA-15 surface. It is noteworthy that the composites maintained excellent mineralization properties even during the manufacturing of various pharmaceutical forms (except for the membrane-type formulation, for which an additional post-manufacturing rapid coating procedure was required), which significantly extends the range of their potential application. Obtained composites showed promising features that respond to the needs of useful dental implant material. In the long-term perspective, it may reveal not only possibly high osseointegration capability but also antimicrobial activity due to the well-documented drug loading capacity of SBA-15 and favored release profile (prolonged with reduced initial burst). Therefore, mesoporous silica-calcium phosphate composites provide the opportunity of their future usage as dual-function addition to dental resins, which are currently applied in dental caries treatment. However, to better characterize and define the specific function of proposed materials in the dental industry, further research in terms of physicochemical properties, drug loading capacity, and biocompatibility are necessary. 

## Figures and Tables

**Table 1:** The ion concentrations of prepared coating solutions with the corresponding reference.

Solution Number	Ion Concentration (mmol/L)								
	Na^+^	K^+^	Mg^2+^	Ca^2+^	HPO_4_^2−^	H_2_PO^4−^	Cl^−^	HCO^3−^	SO_4_^2−^
S1 ^a^ [53]	1013.6	5	5	25	-	3.6	1065	10	-
S2 ^a^ [52]	1010	5	5	25	-	10	1065	10	-
S3 [54]	4	-	-	5	-	2.5	10	1.5	-
S4 ^b^ [51]	710	25	7.5	12.5	5	-	740	21	2.5
S5 ^c^ [55]	142	5	1.5	2.5	1	-	103	27	0.5

^a^ NaHCO_3_ was added to the solution immediately before microwave treatment according to Ref. [53] ^b^ 5 × concentrated SBF (pH = 5.8) [51] ^c^ conventional SBF.

**Figure A1:**
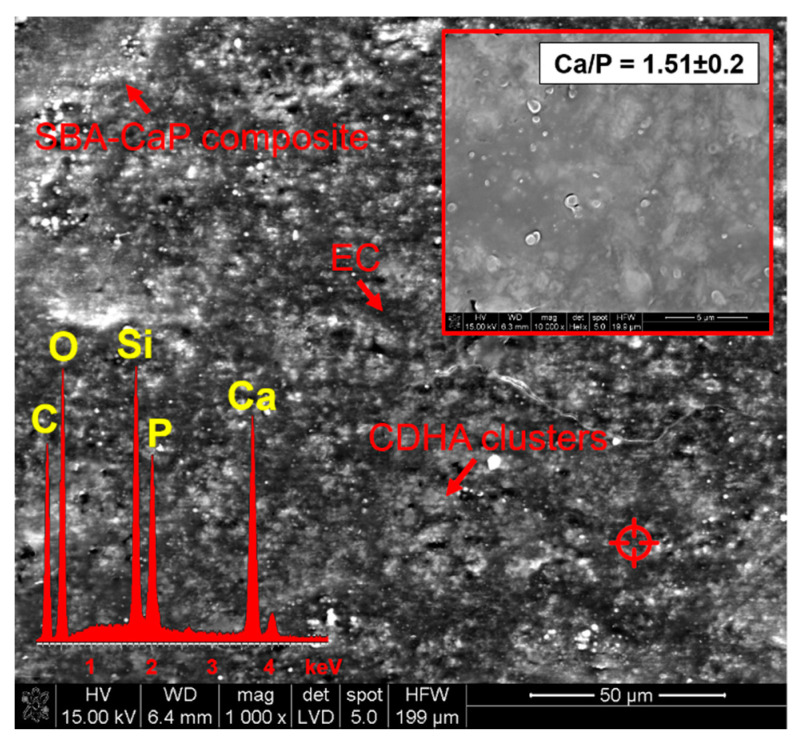
SEM-EDX micrograph of primarily obtained membrane after 21 days of immersion in simulated body fluid.

**Figure A2:**
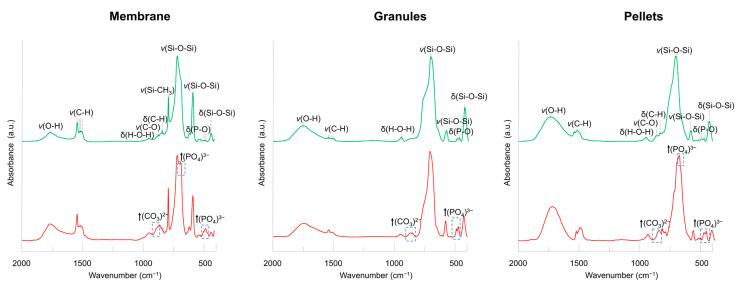
FTIR results of obtained pharmaceutical forms before (green) and after (red) 21 days of immersion in simulated body fluid (types of vibration: ν—Stretching, δ—Bending).

## Data Availability

Data sharing is not applicable to this article.

## Supplementary Materials

The following are available online at https://www.mdpi.com/2073-4360/13/1/53/s1, Figure S1: The flowchart of the study design: SBA-15-calcium phosphate composites synthesis and mineralization properties investigation. Figure S2: FTIR (left) and XRD (right) results of obtained SBA-CaP composites using standard (dashes) and microwave-assisted (straight lines) rapid coating procedures (types of vibration: ν—stretching, δ—bending). Arrows correspond to the vibrational modes characteristic for dicalcium phosphate dihydrate (DCPD). Figure S3: SEM-EDX, FTIR, and XRD results of SBA-CaP(MW)_S3 composite before (green) and after (blue) 7 days of incubation in simulated body fluid with marked areas confirming the poor mineralization potential of obtained composite.
