# Supplementary material for: Microwave-Assisted Fabrication of Mesoporous Silica-Calcium Phosphate Composites for Dental Application
Source: Polymers (Basel). 2020 Dec 25;13(1):53. doi: 10.3390/polym13010053 (PMC7796352; doi:10.3390/polym13010053)
Supplement: Supplementary file 1 [file polymers-13-00053-s001.pdf]

polymers MDPI

Supplementary

Microwave-assisted fabrication of mesoporous silica-calcium phosphate composites for dental application

Adrian Szewczyk [1], Adrianna Skwira [1], Marta Ginter [1,2], Donata Tajer [1,2] and Magdalena Prokopowicz [1,*]

[1] Department of Physical Chemistry, Faculty of Pharmacy, Medical University of Gdańsk, Hallera 107, 80-416 Gdańsk, Poland
[2] Scientific Circle of Students
* Correspondence: magdalena.prokopowicz@gumed.edu.pl

Supplementary Information

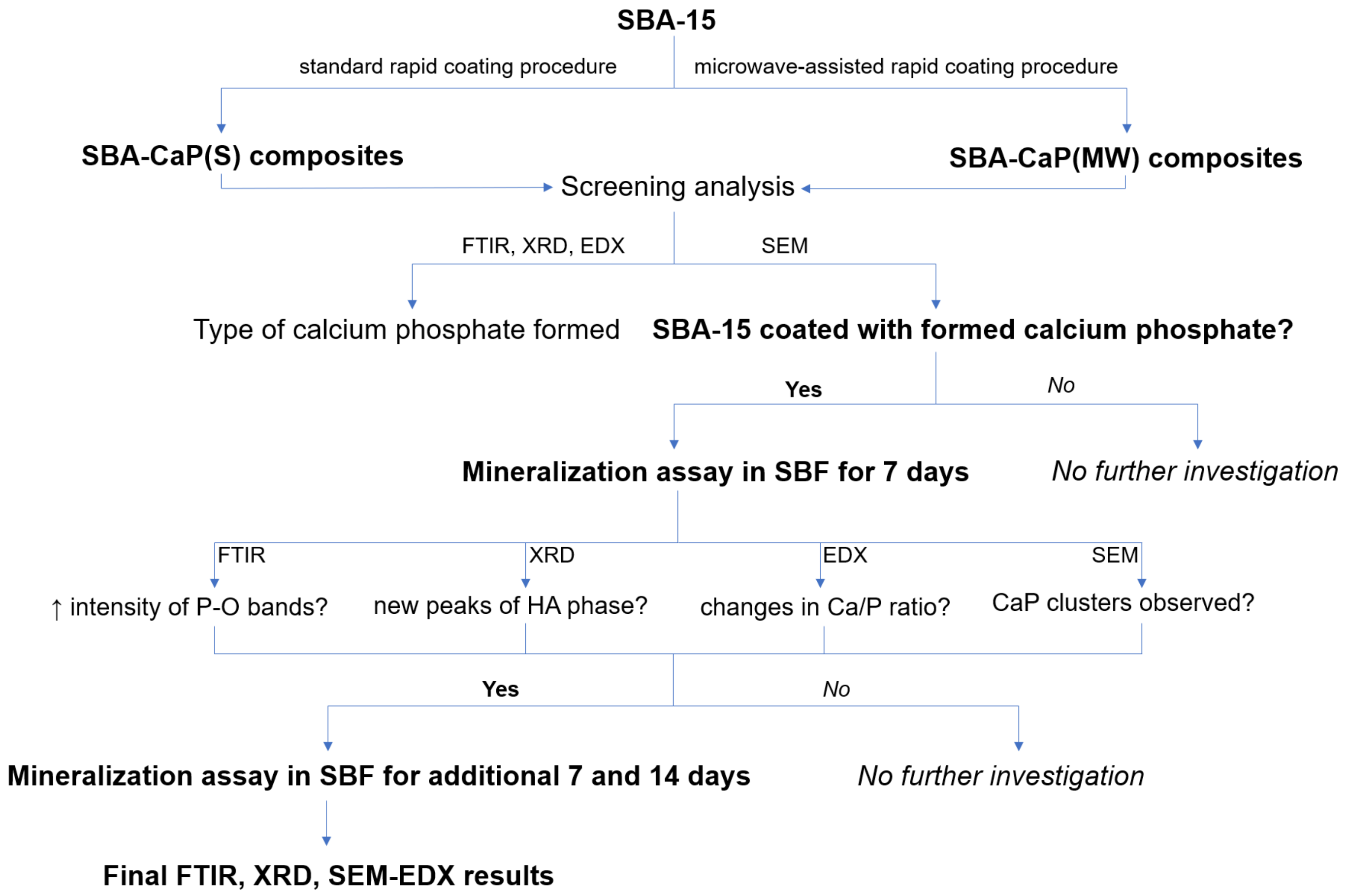

Figure S1. The flowchart of the study design: SBA-15-calcium phosphate composites synthesis and mineralization properties investigation.

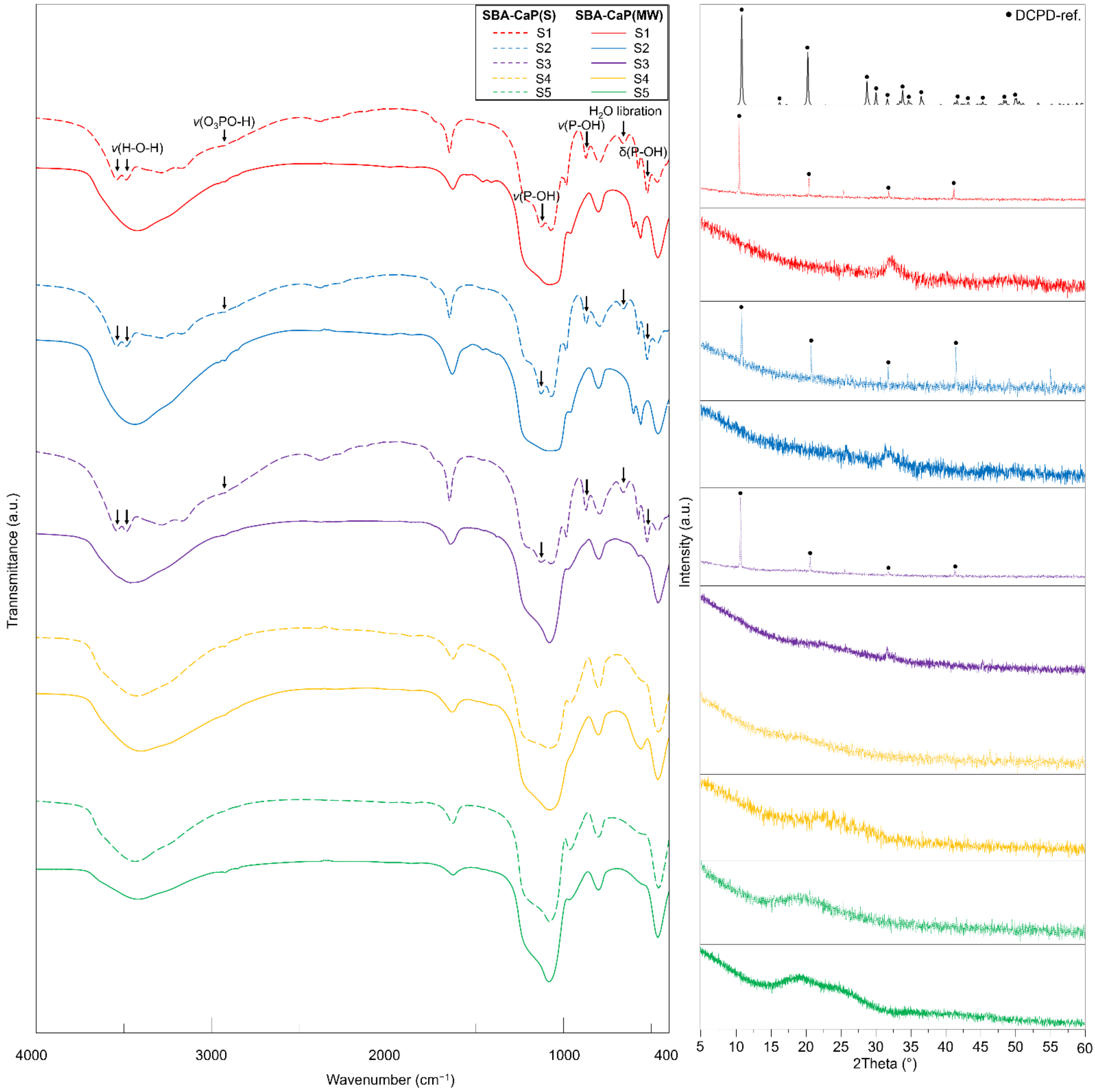

Figure S2. FTIR (left) and XRD (right) results of obtained SBA-CaP composites using standard (dashes) and microwave-assisted (straight lines) rapid coating procedures (types of vibration: ν—stretching, δ—bending). Arrows correspond to the vibrational modes characteristic for dicalcium phosphate dihydrate (DCPD).

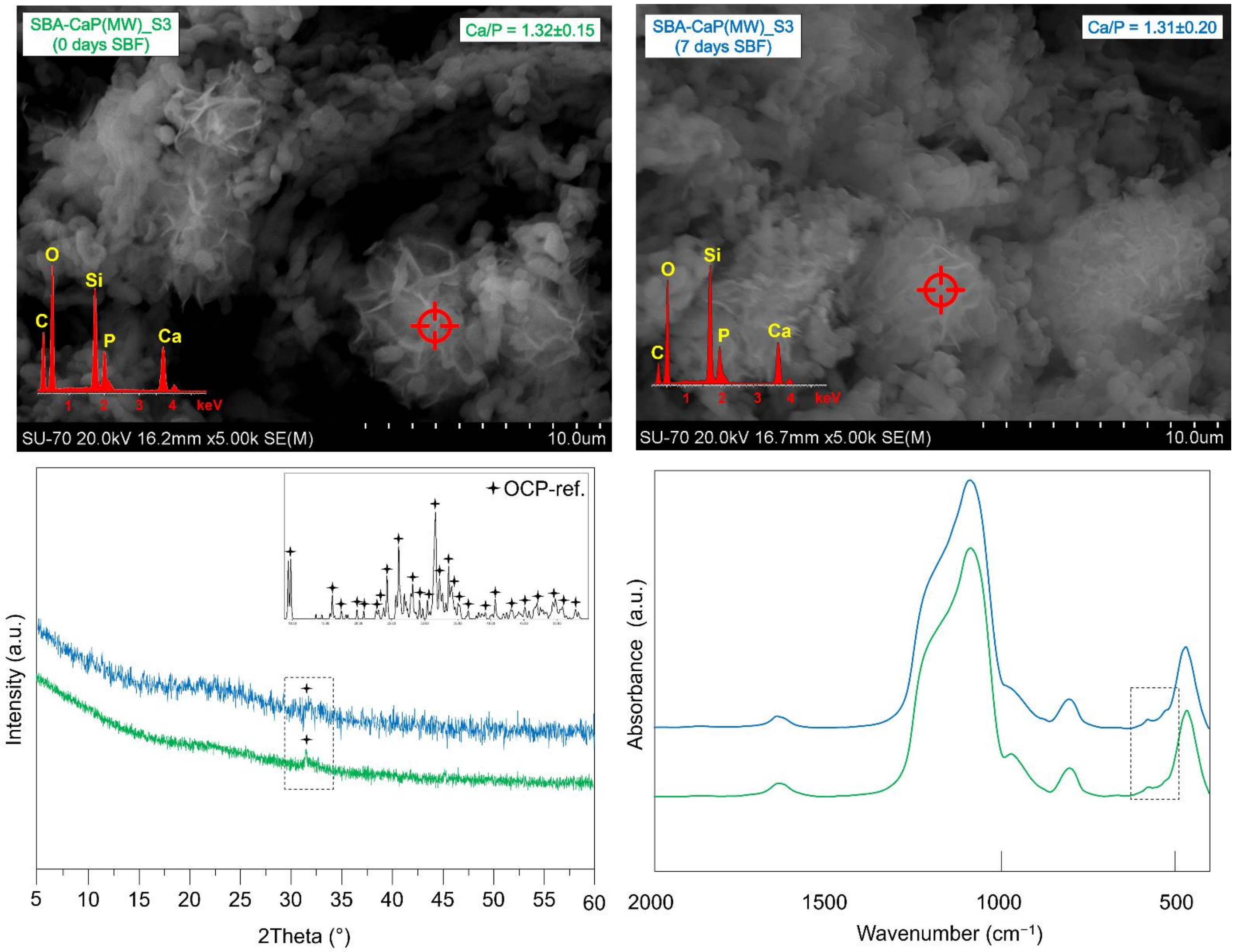

Figure S3. SEM-EDX, FTIR, and XRD results of SBA-CaP(MW)_S3 composite before (green) and after (blue) 7 days of incubation in simulated body fluid with marked areas confirming the poor mineralization potential of obtained composite.
